# Smart-driven bioengineering techniques for enhancing microalgal biohydrogen production

**DOI:** 10.1186/s13068-026-02754-6

**Published:** 2026-03-09

**Authors:** Zhongliang Sun, Shoukai Guo, Adamu Yunusa Ugya, Weixian Cheng, Yu Zhang, Liqin Sun

**Affiliations:** 1 School of Life Sciences, Yantai University, Yantai, China; 2 State Key Laboratory of Crop Stress Adaptation and Improvement, School of Life Sciences, Henan University, Kaifeng, China

**Keywords:** Hydrogenase optimization, Bioreactor design, Renewable energy, Co-product generation, Metabolic reprogramming

## Abstract

Microalgae have the potential to produce hydrogen through photosynthesis, making them a promising alternative to traditional fossil fuels. Although the progress in large-scale production is limited by biological constraints, such as low hydrogen production rates and sensitivity to environmental conditions, the bioengineering of microalgae is an important tool that will help overcome these limitations by enhancing hydrogen production efficiency and improving tolerance to varying environmental conditions. The review indicates the effectiveness of the inhibition of photosystem II (PSII), the introduction of oxygen-tolerant hydrogenase variants, and enhanced electron flow to hydrogenase enzymes as effective strategies to improve hydrogen production in microalgae. The role of integrated systems that combine hydrogen production with co-product generation, such as biofuels, bioplastics, or high-value metabolites, will enhance economic feasibility and sustainability. Also, advancements in bioreactor designs, coupled with real-time monitoring and control systems, create optimized environments that favor large-scale production. This integrated bioengineering approach not only maximizes biohydrogen potential, but also aligns with circular bioeconomy principles by minimizing waste and utilizing resources efficiently. Exploring new ways to enhance the integration of the use of microalgae for biohydrogen production and other valuable products will drive a more efficient and environmentally friendly bioprocess.

## Introduction

The continuous dependence on fossil fuels such as coal, oil, and natural gas as a primary source of energy is associated with issues related to environmental sustainability, economic stability, and human health [1]. Environmental consequences range from climate change, habitat destruction, and negative footprints on natural resources [2]. Economic issues related to fossil fuels include high and volatile production costs, expensive transport and refining, growing regulatory and cleanup expenses, and long-term liabilities that raise energy prices and strain public finances [3]. Furthermore, reliance on fossil fuels is also linked to respiratory and cardiovascular disease, lung cancer, and premature mortality due to harmful air pollutants, disproportionately affecting vulnerable populations [4]. These impacts highlight the interdependence of environmental, economic, and human health concerns, thus buttressing the need for a transition to renewable energy sources like solar, wind, hydroelectric power, and bioenergy [5]. Different types of bioenergy alternatives, such as biofuels and biogas, have been shown to be viable options for reducing greenhouse gas emissions and promoting sustainable energy production [6]. These bioenergy alternatives are produced from sources such as agricultural waste, forestry residues, and organic municipal waste, which have been linked to different limitations [7]. The fact that bioenergy sources depend on factors such as land use practices and resource availability buttressed the need for sources of bioenergy that do not create competition with food production for land and water resources [8].

The surge in the need for sustainable energy alternatives has shifted focus toward microalgae-based biohydrogen [9]. This is alluded to the ability of microalgae to produce hydrogen gas through the process of photosynthesis [10]. This method offers a pathway toward environmental sustainability, thus eradicating issues related to greenhouse gas emissions and dependence on finite fossil fuels [11]. This is attributed to the unique characteristic of microalgae in utilizing sunlight and CO_2_ for photosynthesis, resulting in rapid growth and high productivity. The ability to rapidly grow on non-arable land, needing minimal freshwater, and integrating with wastewater treatment also makes microalgae a sustainable and environmentally friendly option for biohydrogen production. Additionally, dependence on microalgae resources eradicates food–fuel competition, induces the reduction in lifecycle emissions, enables continuous production, and leverages high photosynthetic efficiency with low input requirements [9]. This unique characteristic decreases the competition of freshwater with agriculture or drinking supplies, making microalgae a more sustainable option for biohydrogen production in the long term [12]. Also, the dependence on microalgae increases carbon neutrality over other biomethods because microalgae actively absorb carbon dioxide during photosynthesis, reducing the concentration of carbon footprint [13]; whereas, other biomethods, such as fermentation, lack this carbon sequestration ability, thus increasing carbon footprint in the overall process [14]. Also, the use of microalgae for biohydrogen production does not directly compete with food production because the cultivation can occur in non-arable land, bioreactors, ponds, or wastewater [15].

Despite the advantage of using microalgae as a resource for biohydrogen production, challenges such as low efficiency, limited hydrogen yields, and high susceptibility to environmental stress have hindered its commercial viability [16]. It is thereby important to develop an effective method of counteracting these challenges to enhance the economic feasibility of microalgae-based biohydrogen production [17]. Synthetic biology is an emerging field that can be used to eradicate these challenges, thus enhancing the biohydrogen potential of microalgae resources [18, 19]. This field combines the principles of engineering and biology to optimize enzyme activity and reprogram metabolic pathways in microalgae for increased biohydrogen production [20]. The engineering of microalgae tends to improve light absorption, hydrogenase activity, and electron transport efficiency, thus maximizing biohydrogen production [21]. These tools can also be used to enhance microalgae tolerance toward stressful environmental conditions, further increasing their biohydrogen potential [22]. The use of synthetic biology tools such as CRISPR/Cas9 genome editing eases targeted modification of microalgae, thus enhancing biohydrogen production potential [23].

This review provides a comprehensive perspective on how smart bioengineering and synthetic biology strategies can be employed to enhance the biohydrogen potential of microalgae. It focuses on the unique physiological and metabolic features that make microalgae a sustainable powerhouse for both bioenergy generation and environmental protection. Specifically, the review highlights microalgal traits such as high photosynthetic efficiency, rapid growth rate, and the ability to utilize waste streams such as CO_2_ emissions and wastewater for biomass accumulation. In addition, it summarizes the latest advancements in synthetic biology approaches aimed at improving the suitability of microalgae for biohydrogen production. Finally, the review explores the emerging integration of AI-driven strain optimization, which offers a predictive framework for identifying ideal genetic modifications to achieve maximal H_2_ output.

## Key bottlenecks in microalgal photobiological hydrogen production

Several challenges, such as low yield, oxygen sensitivity, energy inefficiency, and scale-up production issues, limit the application of microalgae as a source of bioenergy production [24]. The low yield of biohydrogen poses limitations to the application of microalgae as a source for biohydrogen production [25]. This limitation affects the economic viability of the process because low yields increase the cost per unit of biohydrogen produced and also extend production cycles, leading to poor return on investment (ROI) [26]. This poor ROI is attributed to the large-scale infrastructural facilities requirement, such as advanced photobioreactors and nutrient supply systems. The intensity of resources needed for microalgae cultures also hinders the yield of biohydrogen production, making it a less attractive option for investors looking for quicker ROI [27]. Oxygen sensitivity also affects the progress of microalgae as a source of biohydrogen due to the fact that oxygen inhibits the enzyme responsible for hydrogen production [28]. For example, the change in oxygen level in the microalgae system tends to cause the inactivation of hydrogenase enzymes, leading to a loss of functionality [29]. This loss of functionality affects the efficiency of the microalgae system to produce biohydrogen, leading to a low yield of hydrogen. These buttress the need to control oxygen levels in the microalgae system in order to optimize biohydrogen production [30].

Energy inefficiency also hindered the progress of microalgae as a source of biohydrogen because the high energy input required for cultivation and extraction processes outweighed the potential benefits of biohydrogen production [31]. Particularly with the fact that the maintenance of a microalgae cultivation system requires the maintenance of optimal conditions such as light intensity, temperature, and CO_2_ levels, which require high energy input [32]. Other issues hindering large-scale production include low and unstable hydrogen yields due to oxygen-sensitive hydrogenases, culture contamination, high costs of photobioreactors, energy-intensive harvesting, and strain instability under variable conditions [33]. Large-scale cultivation is faced with challenges such as uneven light distribution and oxygen accumulation, leading to high operational costs due to the need for higher energy requirements to maintain optimal conditions for growth throughout the entire volume, ensuring efficient mixing and gas transfer [34]. Scale-up cultivation of microalgae is also prone to contamination risks because of the larger volumes of media and equipment involved, increasing the chances of introducing unwanted microorganisms into the culture. This tends to disrupt the biohydrogen production potential of the system due to the competition for nutrients and resources between the desired microalgae and contaminants [35]. The supply and recycling of nutrients are also a limiting factor for large-scale microalgae-based biohydrogen production. This is because the nutrients required for microalgae growth, such as nitrogen and phosphorus, need to be carefully managed to prevent eutrophication of water bodies [36]. The recycling of nutrients from spent biomass also poses some limitations in large-scale production because of the challenges in efficiently extracting and reusing these nutrients [37].

## Bioengineering strategies to improve microalgal biohydrogen yields

Microalgal biohydrogen production via photobiological pathways remains fundamentally limited in conventional wild-type strains due to oxygen inhibition of hydrogenase enzymes and intrinsic metabolic bottlenecks. Recent analyses report volumetric production rates typically ranging from ~ 5–30 mL H_2_ L^−1^ h^−1^ under optimized laboratory conditions for green microalgae such as *Chlamydomonas reinhardtii* and *Scenedesmus obliquus*, with corresponding light-to-H₂ energy conversion efficiencies of < 1% in wild-type systems [38]. Other controlled experiments indicate yields of ~ 0.015–1 mmol H_2_ L^−1^ h^−1^ depending on light, nutrient, and sulfur deprivation strategies. These rates are orders of magnitude below industrial benchmarks (e.g., electrolytic H_2_ production > 1 mol H_2_ L^−1^ h^−1^), underscoring the urgent need for bioengineering to elevate productivity [39]. Microalgal H₂ production is highly sensitive to environmental and nutritional parameters. Key reported conditions include CO_2_ levels, nutrient levels, illumination, and photoperiod. Cultures with elevated CO_2_, such as industrial flue gas mixtures up to 10–20%, have shown increased biomass and thus latent H₂ production capacity [40]; whereas, sulfur deprivation has been found to enhance H₂ production in *Chlamydomonas* due to a reduction in O_2_ evolution and activation of hydrogenases, while balanced nitrogen and trace elements (Mg, Zn) have been linked to biomass growth and enzyme maintenance [41]. Photobioreactor geometry and scale have also been shown to influence light distribution, mass transfer, and scalability. Flat-panel and tubular photobioreactors are preferred for laboratory and pilot scales due to high surface-to-volume ratios that minimize light shading and improve photon utilization. Transparent geometries allow uniform illumination and help maintain micro-oxic conditions essential for H_2_ evolution. Scale remains largely bench-scale (< 10 L) with few validated industrial prototypes, largely due to compromised light penetration and O_2_ accumulation at larger scales, which deactivate hydrogenase [22]. Meeting the milestones where microalgae biohydrogen competes with electrolytic or steam-reformed H₂ demands integrated bioengineering strategies such as metabolic and synthetic biology to upregulate hydrogenase and redirect electron flow, advanced reactor materials and light management, and process control to sustain prolonged H₂-producing states [42].

Bioengineering of microalgae for enhanced biohydrogen production is achieved using different genetic engineering tools, such as CRISPR–Cas9 and TALENs, to modify the genetic and metabolic pathways in microalgae [43]. These modifications can increase the expression of hydrogenase enzymes and improve the overall metabolic pathways involved in biohydrogen production as shown in Table 1 [44]. These tools can also ease the process of developing microalgae strains that are more resilient to environmental stressors, leading to higher biohydrogen yields [45]. The optimization of biohydrogen yield in microalgal systems can be achieved through the modification of the activities of hydrogenase leading to the enhancement of the tolerance of hydrogenase to oxygen, and enhancement of electron flow to hydrogenase [46].

**Table 1:** Synthetic and genetic engineering strategies enhancing microalgal biohydrogen production

Microalgae species	Engineering strategy	Improvement in biohydrogen production	References
*Chlamydomonas reinhardtii*	Site-directed mutagenesis in mitochondria and chloroplasts	Targeted mutations at mitochondrial complex I and PGRL1 (double amino acid deletions at residues 239–241) increased H_2_ yield to 170 ± 2, 140 ± 2, and 150 ± 2 mL H_2_ L^−1^ under TAP-TAP(S) conditions	[47]
*Chlorella* sp.	Engineering of hydrogenase to prevent O_2_ from reaching the enzyme active site	The engineered microalgae produce more biohydrogen that is sevenfold higher than the wild type when exposed to 5% of O_2_ during biophotocatalysis	[48]
*Synechocystis* sp. PCC 6803	Introduction of the O_2_ tolerant hydrogenase complex from *Cupriavidus necator* (*Cn*SH)	Expression of CnSH increased H_2_ output threefold relative to wild type	[49]
*Chlorella* sp. DT mutants	Overexpression of ferredoxin 1 from *Chlamydomonas reinhardtii* (CrFd1, encoded by *crfd1*)	Mutants DT-crfd1-4, DT-crfd1-22, and DT-crfd1-23 showed 4.4-, 5.0-, and 3.8-fold higher H₂ production, respectively, than wild type	[50]
*Chlamydomonas reinhardtii*	Proton-gradient regulation 5 (PGR5)-deficient mutant	PGR5-deficient mutant exhibited a 2.5-fold increase in H_2_ yield compared with wild type	[51]
*Chlamydomonas reinhardtii*	Knock-Down of isoflavone reductase (IFR1) protein	IFR1-knockdown strains showed prolonged H₂ evolution, with final yields 68 ± 10% (IFR1-1) and 93 ± 12% (IFR1-6) higher than wild type	[52]
*Chlamydomonas reinhardtii*	Fusion of ferredoxin and HydA in expression in the model organism	Fusion of ferredoxin and HydA enhanced H₂ production by 4.5-fold over wild type	[53]
*Chlamydomonas reinhardtii*	Engineering of clostridial [FeFe]-hydrogenase and expression in the chloroplast genome of the microalgae	Mutant variant displayed improved O_2_ tolerance and higher H_2_ yield than wild type	[54]

### Engineering hydrogenases to overcome oxygen sensitivity

**Fig. 1:**
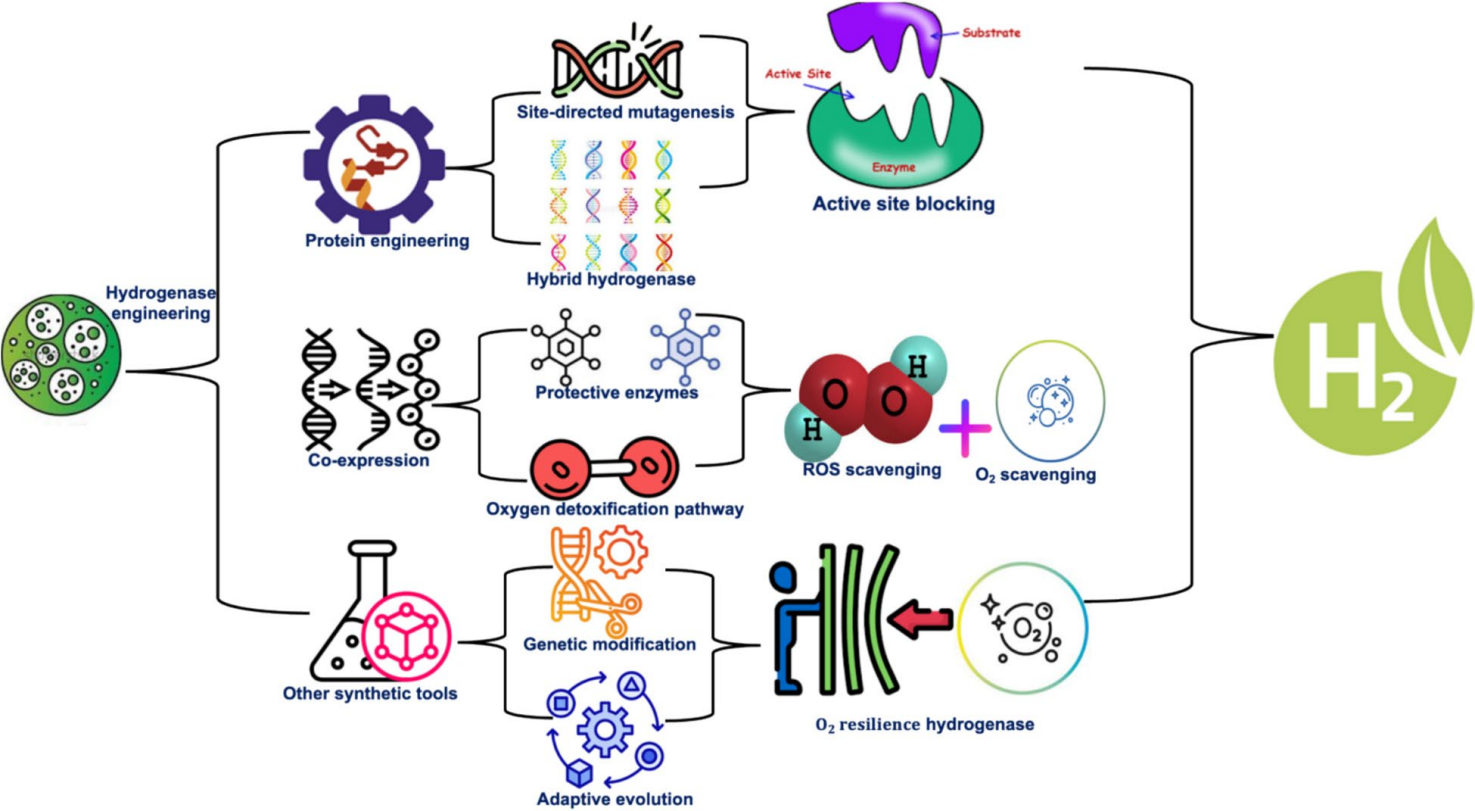
Synthetic and genetic engineering strategies for overcoming the oxygen sensitivity of microalgal hydrogenases

The activities of hydrogenase can be enhanced, leading to increased resistance to environmental stresses and improved hydrogen production rates [55]. The activities of hydrogenase in microalgae are enhanced by precise or random genetic modifications to enhance oxygen tolerance as shown in Fig. 1. This mutagenic modification can be achieved through various techniques, such as site-directed mutagenesis or directed evolution [56]. These techniques can be used for the creation of molecular shields around the active site, thus preventing oxygen from reaching the substrate and causing unwanted oxidation reactions [57]. Cano et al. reported that the target mutation of the I64M of the *Synechocystis* sp*.* hydrogenase causes alteration in the gas diffusion kinetics, thus improving its tolerance to O_2_ [58]. Directed evolution involves the modification of the enzyme's genetic sequence to enhance its catalytic activity. This is achieved through multiple rounds of mutation and selection, resulting in an enzyme with improved efficiency and stability [59]. Similarly, the synthesis of chimeric hydrogenases through the fusion with oxygen-tolerant domains from one hydrogenase with the active site of another, leading to an increase in the catalytic activity of the enzyme in the presence of oxygen. Plummer et al. created 113 hydrogenase gene variants from the combination of hydrogenase from *Chlamydomonas reinhardtii* and *Scenedesmus obliquus,* leading to the screening of mutants that produce hydrogen gas that is 2–3 times higher than the wild-type hydrogenase [60]. The active site optimization of microalgae hydrogenase involves the modification of the enzyme's structure to better accommodate substrate binding and electron transfer, leading to increased efficiency in hydrogen production. This process typically involves site-directed mutagenesis to alter specific amino acids within the active site, as well as the incorporation of cofactors or metal ions to enhance catalytic activity [61]. Sybirna et al. reported that the replacement of aspartic acid in place of arginine^171^ induced an increase in the catalytic activity of *Chlamydomonas reinhardtii* [Fe–Fe] hydrogenase by sixfold compared to the wild type [62]. Site-directed mutagenesis is used for targeted modification of specific nucleotide sequences in the hydrogenase gene to enhance its oxygen tolerance. Engelbrecht et al. using change method causes a site-directed mutagenesis that enhances the contribution of hydroxyl group over threonine 226 in hydrogenase of *Chlamydomonas reinhardtii*. This alters the electronic properties of the active sites leading to a change in the catalytic function of the hydrogenase that enhances its efficiency in producing hydrogen gas [63].

Another important method for increasing the tolerance of microalgae hydrogenase is the incorporation of oxygen-tolerant variants. These can be achieved through the heterologous expression of hydrogenase genes from oxygen-tolerant organisms, such as *Ralstonia eutropha* or other aerobic bacteria. These oxygen-tolerant organisms possess [NiFe]-hydrogenases that are naturally more resistant to oxygen exposure compared to the more commonly used [FeFe]-hydrogenases. So the expression of oxygen-tolerant hydrogenase genes in microalgae can help improve their hydrogen production efficiency under aerobic conditions [64]. Weyman et al. reported that the co-expression of [NiFe] hydrogenases from *Alteromonas macleodii* and *Thiocapsa roseopersicina* in *Synechococcus elongatus* resulted in hydrogen evolution activity when the mutants were tested using an in vitro hydrogen evolution assay [65]. Genomic editing tools such as CRISPR–Cas9 and TALENs can be used to precisely edit genes associated with hydrogenase or its regulators. These tools allow for targeted modifications to increase the expression of oxygen-tolerant variants, leading to enhanced catalytic efficiency and stability in aerobic conditions. Also, the introduction of genetic circuits that sense and respond to oxygen levels by activating protective mechanisms can further improve the performance of hydrogenase in aerobic environments. Hwang et al. reported that the upregulation in the expression level of the hydrogenase gene in *Chlorella vulgaris* produces biohydrogen under high oxygen concentration reaching a maximum concentration of 0.15–0.69% within 7 days of cultivation [66]; whereas, the expression of ferredoxin-hydrogenase fusion enzyme in *Chlamydomonas reinhardtii* by Weiner et al. (2018) triggers an increase in biohydrogen production from 12.6 ± 3.9 to 30 ± 5.9 μmole H_2_ × mg chl^−1^ × h^−1^ that is 4.5-fold higher than the wild type [67]. Wang et al. reported that the aggregation of *Synechocystis* sp. and *Chlamydomonas reinhardtii* using gel immobilization led to an increase in biohydrogen production from 4.4 and 155.7 μmol H_2_/mg chlorophyll, respectively, to 46.3 and 1226.4 μmol H_2_/mg chlorophyll, respectively, amounting to a 10.5- and 7.9-fold increase, respectively. This increase in hydrogenase activities was linked to enhanced physiological properties such as integrity, porosity, and oxygen diffusion coefficient. The aggregation of the microalgae through gel immobilization also promoted hydrogen production increase by 1.89- and 2.02-fold in *Synechocystis* sp. and *Chlamydomonas reinhardtii*, respectively [68].

### Redirecting photosynthetic electron flow toward hydrogenase

**Fig. 2:**
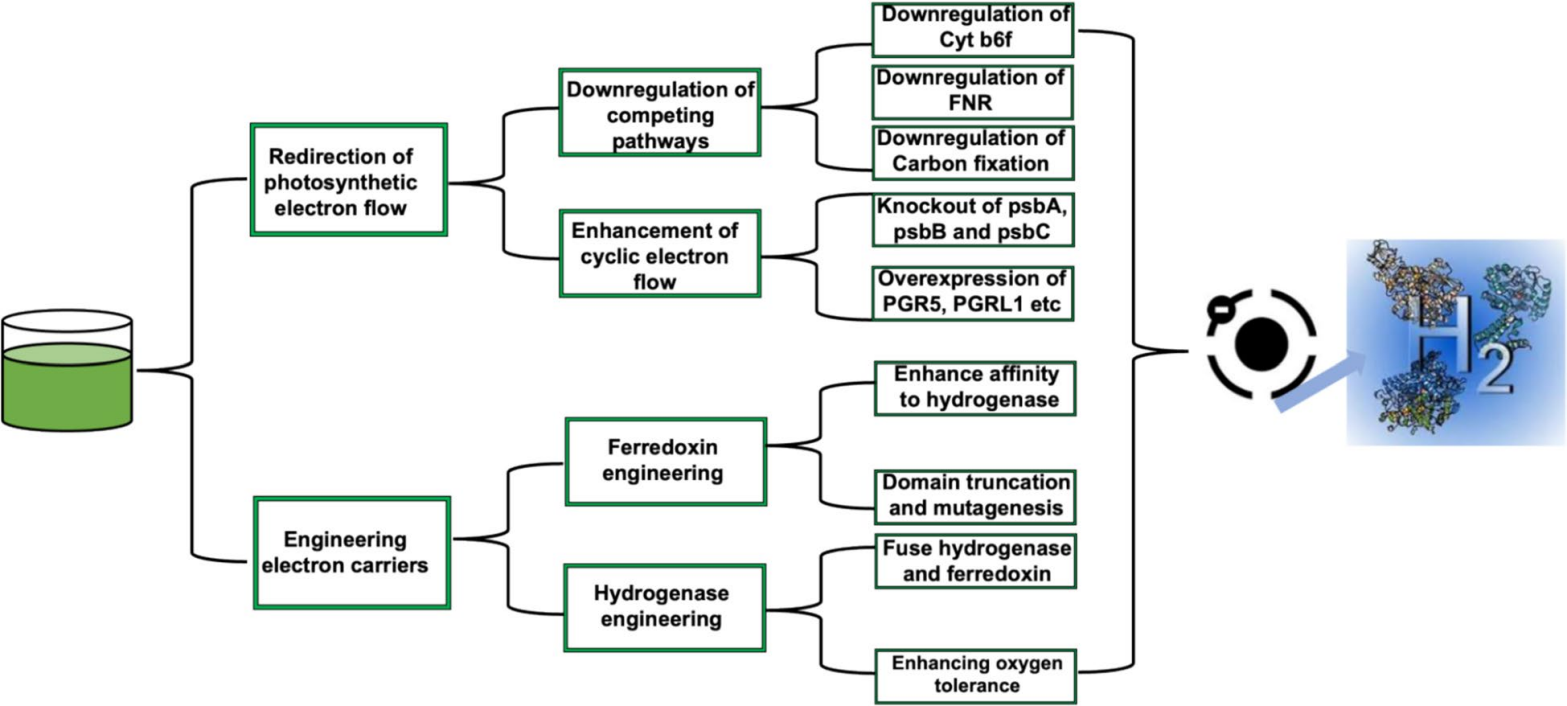
Strategies for enhancing electron flow toward hydrogenase in microalgae

Biohydrogen production efficiency in microalgae can be enhanced by increasing the flow of electrons within the hydrogenase complex. This causes an improvement in the catalytic efficiency of the enzyme, leading to higher rates of hydrogen production [69]. This is achieved through synthetic biology and metabolic channeling techniques, which involve manipulating the metabolic pathways within the microalgae to prioritize hydrogen production. The methods and strategies used to enhance the flow of electrons toward hydrogenase during photobiological H_2_ production in green microalgae include redirection of photosynthetic electron flow and engineering electron carriers as shown in Fig. 2 [70].

The redirection of photosynthetic electron flow in microalgae can be achieved through the knockout or downregulation of competing pathways, and the enhancement of cyclic electron flow (CEF) control. The knockout or downregulation of competing pathways can lead to an increase in redirection of electron flow toward hydrogenase because it reduces the availability of alternative electron sinks [71]. For example, the downregulation of the cytochrome b6f complex (Cyt b6f) can limit the flow of electrons to alternative pathways, allowing for a greater proportion of electrons to be redirected toward ferredoxin reduction for hydrogenase. This is attributed to the fact that the downregulation of the Cyt b6f triggers the partial inhibition of Photosystem II (PSII) in microalgae. The partial inhibition of PSII in microalgae favors the increase in the activities of hydrogenase enzymes, leading to higher hydrogen production rates [72]. This inhibition causes the decrease in the production of oxygen while maintaining Photosystem I (PSI) functionality, thus making more electrons available for hydrogen production [73]. The targeted gene knockout of genes such as psbA, psbB, and psbC using techniques such as RNA interference (RNAi), CRISPR–Cas9, and antisense RNA technology tends to induce the downregulation of Cyt b6f. This is because Cyt b6f depends on reduced plastoquinol (PQH₂) from PSII as its electron source. The knockout of these genes thus favors enhanced biohydrogen production in microalgae due to an increase in the availability of electrons for hydrogenase enzymes that prompt biohydrogen production. For example, Li et al. used artificial miRNA to silence psbA in *Chlamydomonas reinhardtii,* leading to a slightly low cell density and chlorophyll content of 4–5 × 10^6^ cells/mL and 20 mg/L, respectively, compared to the wild type at the 5th day of cultivation. The study also reported a higher yield of hydrogen, attaining a maximum level that is 57.1% (± 28.6%) higher than the wild type [74]. Similarly, the downregulation of carbon fixation in microalgae leads to the availability of more electrons for biohydrogen production. For example, the knockout of Rubisco from microalgal cells tends to increase hydrogen production by microalgal systems because more electrons for carbon fixation are channelled toward hydrogenase enzymes. Similarly, the suppression of the activities of NADPH reductase tends to enhance hydrogen production by redirecting electrons toward hydrogenase enzymes, rather than being utilized in other metabolic processes such as the reduction of NADP⁺ for biosynthesis. Sun et al. [75] reported that the silencing of ferredoxin-NADP^+^ reductase in *Chlamydomonas reinhardtii* using RNAi led to a 60% and 40% reduction in the amount of Rubisco and evolution of photosynthetic oxygen. Thus, signifying the increased flow of electrons to hydrogenase, which favors the photoproduction of hydrogen. The fnr-RNAi mutant strains demonstrated significantly higher H_₂_ production efficiency compared to the wild type under sulfur-deprived conditions. With the maximum production rate achieved at 72 h, the wild type has 37 μl mg⁻^1^ Chl, while the fnr-1 mutant and the fnr-9 mutant have 290 μl mg^⁻1^ Chl h^⁻1^ and 360 μl mg^⁻1^ Chl h^⁻1^, respectively [75]. The downregulation of Ferredoxin: NADP⁺ oxidoreductase (FNR) tends to enhance biohydrogen production in microalgae. This is due to the primary involvement of FNR in the transfer of electrons from ferredoxin to NADP⁺ in the PS I reaction, which competes with hydrogenase for electrons. The decrease in the activities of FNR induces the availability of more electrons for hydrogenase, thus increasing biohydrogen production in microalgae. Sun et al. [75] reported that the downregulation of FNR using RNA interference led to the 44% decrease in the rate of photosynthetic oxygen evolution and 2.5-fold increase in biohydrogen production [75]. This indicates that RNAi-mediated downregulation of FNR triggers the suppression of linear electron flow to NADPH, thereby reinforcing cyclic electron flow (CEF) around PSI via ferredoxin-dependent plastoquinone reduction. The enhancement of CEF causes an increase in proton motive force and ATP synthesis while competitively redirecting the reduction of ferredoxin to [FeFe]-hydrogenase, elevating H₂ yields despite attenuated PSII activity [76]. The enhancement of CEF in microalgae allows for the diversion of electrons from the photosynthetic electron transport chain to produce hydrogen gas. This is because CEF involves the cycling of electrons around PSI via the Cyt b6f and ferredoxin, leading to the generation of ATP without net NADPH production. The process allows for the recycling of electrons from PSI through ferredoxin, PQ, and Cyt b6f, leading to the creation of a proton gradient for ATP synthesis. The use of different genetic engineering methods for the knockout of PSII genes such as *psbA*, *psbB*, and *psbC* leads to an increased reliance on CEF. Kim et al. (2024) reported that the *Chlamydomonas reinhardtii* mutant displaying significantly low PS II activity showed a higher rate of CEF [77]. This indicates that the manipulation of PSII genes triggers the optimization of CEF that triggers an increase in hydrogen gas production in microalgae [30]. Also, the overexpression of CEF-related proteins such as PGR5, PGRL1, or the NDH-2 complex tends to enhance the efficiency of cyclic electron flow (CEF) in photosynthesis, leading to an increase in biohydrogen production in microalgae [78].

The engineering of electron carriers to enhance electron flow to hydrogenase is another important strategy for enhancing hydrogen production efficiency in microalgae. This is achieved through hydrogenase and ferredoxin engineering, which involves modifying the electron transfer pathways to optimize the efficiency of electron transport [79]. For example, engineering hydrogenase to improve electron flow involves the optimization of both the enzyme's intrinsic properties and its integration with electron carriers or electrode materials. The enhancement of the active site and stability of hydrogenase tends to increase electron transfer. This is because the active sites play a crucial role in catalyzing the reaction, while improved stability ensures a longer lifespan for the enzyme, allowing for sustained electron transfer efficiency. So the replacing of cysteine with selenocysteine in [NiFe]-hydrogenases tends to enhance the proton-coupled electron transfer (PCET) efficiency, leading to higher biohydrogen production activity [80]; whereas, Romig et al. (2025) reported that glutathione tends to enhance the long-term stability of the [NiFe] Hox hydrogenase of *Synechocystis* sp. PCC6803 exposed to oxygen [81]. The engineering of ferredoxin also tends to enhance electron flow to microalgae hydrogenase. This is achieved through the optimization of the redox properties, structural interactions, and integration of ferredoxin with hydrogenase systems. The structural modifications of ferredoxin can be attained through [2Fe–2S] cluster engineering and domain truncation and mutagenesis. The [2Fe–2S] cluster engineering involves altering the amino acid residues surrounding the cluster to improve electron transfer efficiency. Wiegand et al. reported that the engineering of the ferredoxin and FDR affinity in *Synechocystis* PCC 6803 enhanced the flow of electrons from ferredoxin to hydrogenase by an 18-fold increase [82]. Also, domain truncation and mutagenesis can help to remove unnecessary structural elements and enhance the overall stability of the protein, ultimately leading to improved electron flow to microalgae hydrogenase. For example, the use of site-directed mutagenesis allows for the modification of amino acid residues in D1 or D2 proteins to reduce PSII efficiency without completely inhibiting the photosystem [46]. Khaing et al. [83] triggered a directed mutagenesis in *Synechocystis* sp. by replacing the *psbDI/psbC* operon encoding D2 and CP43 with a chloramphenicol-resistance cassette, while *psbDII*, encoding a second copy of D2, was replaced with a kanamycin-resistance cassette, leading to a reduction in the electron transfer between PS II and exchange between plastoquinol molecules [83]; whereas, random mutagenesis allows the use of mutagenic agents such as ethyl methanesulfonate (EMS) or UV irradiation to induce random mutations in PSII-related genes. Lai et al. [84] using UVR stress causes a DNA mutation in *Chlorella sp.* that triggers a significant photosynthetic dysfunction leading to a deduction in photosynthetic light harvesting efficiency by between 20.2% and 55.0% depending on strain [84]; whereas, Thurakit et al. [85] performed ethyl methanesulfonate-induced mutagenesis on *Botryococcus braunii* leading to an increase in the biofuel potential of the microalgae [85].

## Integrated systems for sustainable microalgal biohydrogen production

**Fig. 3:**
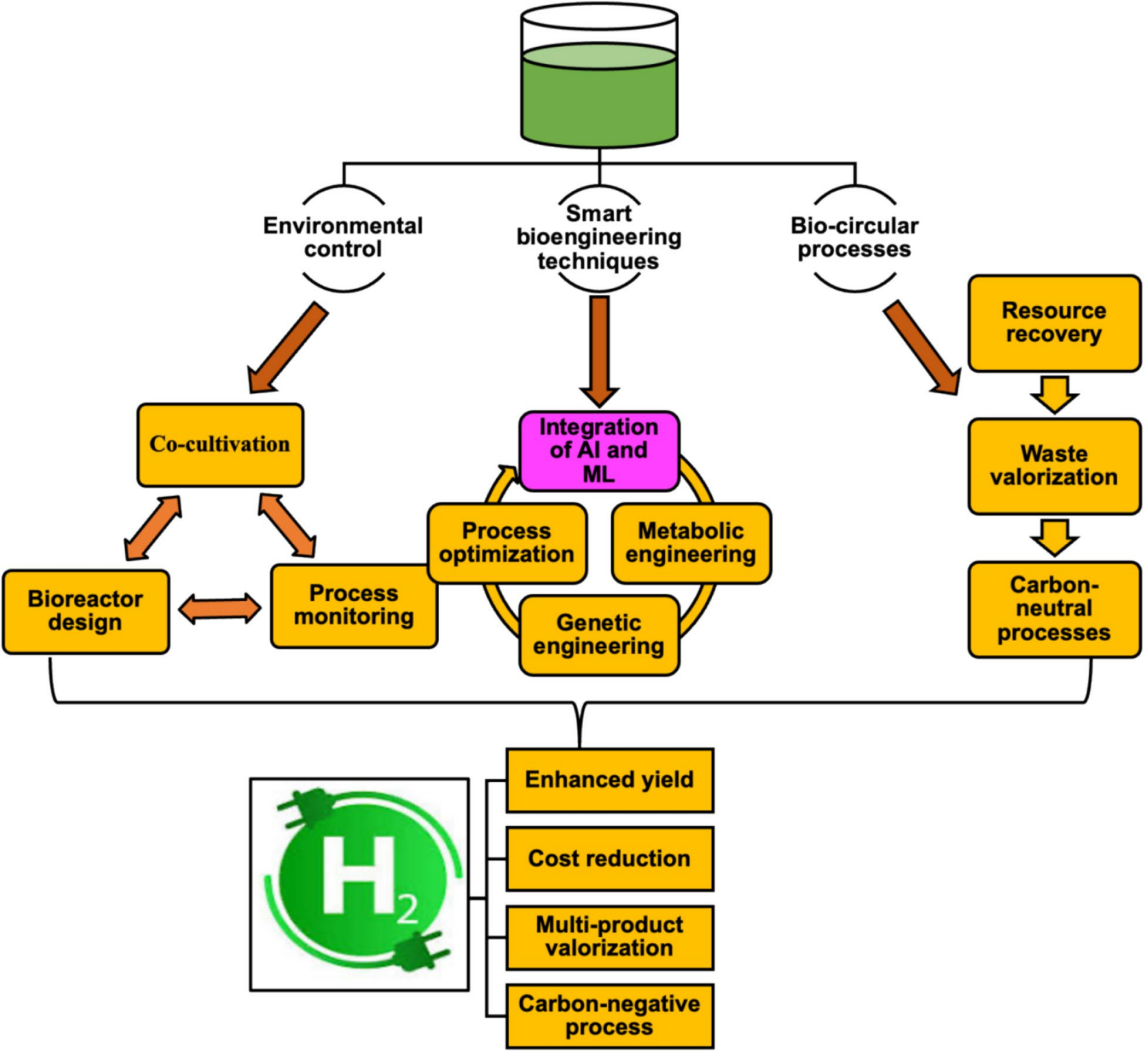
Enhancing the sustainability of microalgae biohydrogen through integrated systems

The bioengineering of microalgae can be economically feasible for biohydrogen production when simultaneously used for the production of valuable byproducts like lipids or pigments to reduce production costs [86]. This dual-purpose approach allows for a more sustainable and cost-effective production process, making biohydrogen production from microalgae a promising renewable energy source for the future [87]. Although co-production with lipids/pigments is constrained by phase antagonism because H₂ evolves under sulfur deprivation and low PSII flux, whereas lipid and carotenoid accumulation require carbon-rich, oxidative states. So the co-production at high yields in a single metabolic state is not intrinsically feasible due to metabolic antagonism. But the use of dynamic cultivation strategies such as two-stage processes, genetic/metabolic rewiring that decouples pathways, or engineered cyclic electron flow to balance ATP/NADPH demand and redirect reductant toward both H₂ and targeted metabolites can potentially overcome these constraints and enhance co-production efficiency. By optimizing cultivation conditions and metabolic pathways, it is possible to achieve higher yields of both H₂ and lipids/pigments simultaneously [88]. Similarly, the bioengineering of microalgae to thrive on industrial or agricultural waste streams as a nutrient source also improves economic viability. This co-processing allows for resource optimization because it reduces the need for costly nutrient inputs and minimizes waste disposal costs [89]. Also, the market demand for bioactive compounds and high-value products derived from microalgae further enhances the economic potential of biohydrogen production [90]. The approach used for the bioengineering of microalgae through integrated system to enhance biohydrogen production involves process optimization techniques such as environmental control, smart bioengineering techniques, and biocircular processes as shown in Fig. 3.

### Process optimization and circular-bioeconomy pathways

Environmental control of microalgae cultivation system involves the optimization of microalgae cultivation systems by integrating various engineering, biological, and environmental factors to maximize efficiency and yield of biohydrogen. The various strategies utilized in the process optimization of microalgae cultivation systems for biohydrogen production and other high-value products include process optimization, co-cultivation, and bioreactor design [91]. Process optimization is critical for enhancing the biocircular economy because it directly increases microalgae biomass yield and biohydrogen production while reducing resource inputs, costs, and environmental impacts. By fine-tuning cultivation parameters such as light intensity, photoperiod, nutrient composition, temperature, pH, and carbon dioxide supply, microalgal growth rates and photosynthetic efficiency can be maximized (Table 2). Leong et al. [92], using optimal nitrate concentrations, produce the maximum carbohydrate productivity of 451.9 ± 16.2 mg/L/day with 65.4% carbohydrate content at an initial nitrate of 0.225 g/L; whereas, the highest biomass productivity at 882.4 ± 13.5 mg/L/day with CO₂ fixation of 9.3 g/L was achieved at an initial nitrate of 0.375 g/L. Continuous feeding with 0.225 g/L initial nitrate peak carbohydrate productivity to 618.3 ± 14.3 mg/L/day and 73.7% carbohydrate content while CO_₂_ fixation was enhanced: from 3.4 g/L to 12.7 g/L when a fed-batch strategy was also employed. The biohydrogen production rate was a maximum H₂ yield of 1.30 mol H_₂_/mol glucose and a productivity of 28.49 mL/L/h with 1:3 algae/glucose as the optimal substrate ratio. While peak H₂ productivity of 312 mL/L/h with 58.9% hydrogen concentration was achieved at 12 h HRT, and pH was maintained at 5.5 throughout cultivation during the continuous fermentation process [92]; whereas, Corrêa et al. [93] reported that the use of exhaust gases from diesel engines in the cultivation of the microalgae *Acutodesmus obliquus* enhanced biohydrogen production and also favored the increase in microalgae biomass from 0.29 ± 0.03 g/L to 1.66 ± 0.04 g/L, 2.04 ± 0.05 g/L and 1.78 ± 0.01 g/L, respectively, depending on emission sources, leading to a cumulative increase of up to 285% in the total lipids [93]. Also, the use of coculture systems to introduce organisms that utilize oxygen or provide metabolites to enhance hydrogen production by microalgae systems is another effective process optimization strategy to enhance productivity and efficiency. This is because the creation of symbiotic relationships between microalgae and other organisms can help create a more stable and balanced ecosystem, leading to increased overall biomass production and hydrogen yield. The symbiotic system reduces the risk of contamination and improves nutrient cycling within the system that favors the accumulation of high-valued metabolic products while maximizing biohydrogen production. For example, Zidan et al. [94] reported that the coculture of *Chlamydomonas reinhardtii* and activated sludge bacteria mitigates the production of excess oxygen to a level that is 153.2 mL/L over a 6-day cultivation period, while the hydrogen production of the system increases to a maximum level that is 1162 mL/L [94].

**Table 2:** Enhancing microalgae biohydrogen production through process optimization

Microalgae species	Process optimization	Improvement in biohydrogen production	References
*Chlamydomonas reinhardtii. Chlorella sorokiniana*	Cultivated in acetate-enriched wastewater at a light intensity of between 50 and 300 μmol/(m^2^·s)	The average amount of biohydrogen produced was 108 ± 4 μmol/L and 88 ± 7 μmol/L for *Chlamydomonas reinhardtii* and *Chlorella sorokiniana*, respectively, at a light intensity of 100 μmol/(m^2^·s)	[96]
*Chlorella vulgaris* *Scenedesmus obliquus*	Cultivated in urban wastewater supplemented with essential nutrients under control light quality with intensity of 140 μmol/(m^2^·s)	Purple light induces an increase in biohydrogen production to a maximum level that is 204.8 mL H_2_/(L·d) and 39.18 mL H_2_/(L·d) for *Scenedesmus obliquus and Chlorella vulgaris*, respectively	[97]
*Chlamydomonas reinhardtii*	Cultivated in olive mill wastewaters supplemented with tris–acetate-phosphate	The optimized condition favors higher biohydrogen production of 150 ml H_2_ /L compared to the control, which was 100 ml H_2_ /L	[98]
*Arthrospira platensis*	Dark- and photo-fermentation conditions were created using *Clostridium butyricum and Rhodopseudomonas palustris* isolated from anaerobic activated sludge; whereas zeolite was used for the removal of impurities and enhancing the removal efficiency of NH_4_^+^	The dark fermentation favors increased biohydrogen production to a maximum level that is 96.6 ml H_2_/g DW, while the modified zeolite triggers the reduction in the concentration of NH_4_^+^ from 31.6–56.5 mM to 2.2–2.7 mM, amounting to 91.8%–95.8% removal efficiency	[99]
*Chlorella kessleri*	Cultivated in food industrial wastewater supplemented by high levels of carbon dioxide	Biohydrogen production reach a maximum level that is 44.24 mL from 1 L of microalgae suspension	[100]
*Scenedesmus obliquus*	Cultivated under different CO_2_ conditions, leading to a CO_2_ fixation ability of 727.7 mg/(L·d). The biomass was also subjected to different pretreatment processes to enhance biohydrogen production	The process optimization enhanced the biohydrogen yield to a maximum level that is 97.6 mL/g when the biomass obtained is pretreated using the acid-thermal pretreatment method	[101]
*Scenedesmus obliquus*	Dark fermentation was engineered using *Enterobacter aerogenes* and *Clostridium butyricum*	Biohydrogen production reach a level that is 113.0 mL H_2_/g VS_alga_ and 57.6 mL H_2_/g VS_alga_ when *Clostridium butyricum* and *Enterobacter aerogenes* were used for the dark fermentation of *microalgal* biomass of 50.0 g/L and 2.5 g/L, respectively*,* after a 6 h fermentation period	[102]
*Chlorella* sp.	A multi-input and single-output framework was used to control cultivation factors such as sulfur content, biomass concentration and the period of cultivation	The biohydrogen production yield was maximum to a level that is 74.32 mL/g at a biomass concentration, sulfur content, and period of cultivation of 100 g/L, 0.9%, and 95.28 h, respectively	[103]
*Chlorella vulgaris MSU-AGM 14*	*Valoniopsis pachynema* extract was used as a carbon and nitrogen source	The photo biohydrogen production reaches an optimum level that is 0.002 g/(L h)	[104]

The design of bioreactor use in microalgae cultivation is also an important technique toward enhancing microalgae biohydrogen production. Systematic optimization of bioreactor geometry directly influences microalgae biohydrogen production rate. The evaluation of tubular bioreactors with diameters of 7, 8, 9, and 10 cm for H₂ production indicates that the 8-cm diameter reactor produced one liter more H₂ than the 10-cm reactor over four days, despite the latter's larger volume. This demonstrates that optimizing the light path to balance photon penetration against the inhibitory effects of both light saturation and hydrostatic pressure is a critical design principle. Furthermore, a panel-type PBR with a 4-cm light path, designed for a superior surface-area-to-volume ratio, underperformed compared to a tubular counterpart due to inefficient mixing from external circulation, underscoring that an enhanced light path must be coupled with effective mixing to facilitate H₂ degassing. These findings confirm that reactor design is not merely about supporting biomass density but about creating the specific physicochemical microenvironments of light, mixing, and gas exchange that sustain hydrogenase activity [95].

The integration of the principles of circular bioeconomy into the bioengineering of microalgae tends to enhance the sustainability of biohydrogen production. This also enhanced the other bioprospecting potential of microalgae, such as the production of biofuels, bioplastics, and high-value compounds [29]. Thus, increasing the achievement of an integrated system that maximizes the efficiency and economic viability of the process through the optimization of resource use, minimizing waste, and creating value-added products while simultaneously ensuring environmental sustainability. For example, the bioengineering of microalgae for biohydrogen generation triggers the production of biomass after extracting high-value compounds. This biomass can be utilized for other bioprospecting applications, such as other forms of bioenergy production or as a source of nutrients for animal feed [105]. Similarly, the use of wastewater for microalgae cultivation enhanced nutrient removal in wastewater. This also signifies the circularity of the process because it increases the potential of microalgae for biohydrogen production and other valuable byproducts. Venkatesh et al. [106] reported that the cultivation of *Chlorella vulgaris* in textile wastewater under the influence of silicon dioxide nanoparticles as a catalyst triggers an increase in hydrogen yield to 66.7 mol/kg, which has a better gasification efficiency of 63.4% and an optimum hydrogen efficiency of 66.8% compared to others [106]; whereas, Ruiz-Marin et al. [97] reported that the cultivation of *Chlorella vulgaris* and *Scenedesmus obliquus* in urban wastewater by inducing sulfur starvation and differences in light quality resulted in better growth of the microalgae under blue light compared to purple light. But the maximum hydrogen productivity of 204.8 mL H_2_/(L·d) and 39.18 mL H_2_/(L·d) was recorded under purple light for *Scenedesmus obliquus* and *Chlorella vulgaris*, respectively [97].

### AI-enabled, data-driven bioengineering integration

The integration of artificial intelligence (AI) and machine learning (ML) into bioengineering is an emerging trend that will enhance microalgal biohydrogen production potential [107]. This tool will ease the optimization of genetic, metabolic, and operational parameters, thus increasing the efficiency and scalability of biohydrogen production processes [108]. AI and ML can also be used as a tool for photobioreactor design optimization, data-driven prediction modeling, life cycle and sustainability analysis of microalgae biohydrogen potential [109].

The use of AI and ML in predictive modeling tends to ease the analysis of large datasets from omics technologies such as genomics, transcriptomics, proteomics, and metabolomics, allowing for more accurate predictions and insights into the complex interactions within microalgae. This eases the identification of key genetic and metabolic targets for enhancing hydrogen production [110]. For example, ML models can be used to predict which genes or pathways associated with hydrogenase enzymes and photosynthetic machinery are most important for biohydrogen production. Thus, easing the targeted genetic engineering efforts to improve hydrogen production in microalgae [111]. Machine learning models enable the design of synthetic genetic circuits and pathways to enhance hydrogen production, for example, by engineering oxygen-tolerant hydrogenases or stress-inducible promoters. These models can predict circuit behavior under various conditions, allowing for optimization prior to experimental implementation. For example, Anuntakarun et al. [112] introduce the microalgae SMOTE Random Forest Relief model (mSRFR) to accurately classify noncoding RNAs to about a level that is 97% and a false-positive rate of about 2%. The classification of noncoding RNAs is significant because it eases the understanding of the diverse functions and roles in gene regulation and cellular processes. Also, accurate classification of noncoding RNAs can lead to the identification of potential targets for genetic engineering to enhance bioproduction processes [112]. Also, AI can be used to simulate and predict the effects of genetic modifications, such as redirecting carbon flux toward hydrogen production or knocking out competing pathways. Thus streamlining the process of developing genetically modified microalgae strains optimized for biohydrogen production. This approach can efficiently accelerate research and development efforts in the field of bioenergy, leading to more sustainable and efficient methods for producing hydrogen fuel [113].

Smart biomonitoring can also be used for the real-time control of microalgae cultivation systems to surge biohydrogen production [114]. This involves the integration of AI-powered systems that have sensors and IoT (Internet of Things) devices to monitor and control bioreactor conditions in real time. The function of the AI-powered system includes dynamic adjustment of parameters, which involves the optimization of light intensity, temperature, pH, and nutrient supply to maximize hydrogen yield. Baicu et al. [115] reported that sensors embedded with IoT systems monitor and transmit data in real time to a cloud platform, thus easing the process of microalgae cultivation and optimizing growth conditions [115]. Another function of AI-powered systems is the early detection of stress through the use of ML algorithms to detect early signs of stress, such as nutrient limitation and oxygen accumulation. This early warning system allows for timely intervention to prevent a decrease in biohydrogen production [116]. The use of smart hybrid systems can also be used to optimize the design and operation of microalgae–bacteria consortia, leading to enhanced hydrogen production. The smart system incorporation eases the prediction of the most effective bacteria for the symbiotic relationship with microalgae, ultimately increasing the overall efficiency of hydrogen production [117]. This increases the functional role of the bacteria in the consortia and allows for more precise control over the production process. The smart hybrid systems integration also helps minimize resource consumption and waste generation, making the production of hydrogen more sustainable in the long run [117].

AI and ML also play a critical role in photobioreactor design optimization of microalgae cultivation systems to enhance biohydrogen yield. This tool can assist in the identification of optimal operating conditions for maximizing microalgae growth and biohydrogen production. For example, computational fluid dynamics (CFD) can be used to optimize light distribution and nutrient mixing in the photobioreactor to ensure efficient photosynthesis and nutrient uptake by the microalgae; whereas, AI can be used in designing smarter reactor geometries and configurations that enhance uniform illumination and gas exchange within microalgae systems [118]. Belohlav et al. [119] reported the use of CFD in the optimization of a pilot hybrid tubular photobioreactor through hydrodynamics analysis, leading to enhancement in microalgae cultivation and wastewater treatment [119]; whereas, Zhao et al. [120] reported that the use of CFD techniques in a wave-driven floating photobioreactor enhanced the efficiency of microalgae cultivation by providing continuous mixing and nutrient distribution, resulting in increased biomass productivity and improved aquaculture wastewater treatment capabilities [120]. The use of AI in the creation of strong predictive models for hydrogen output based on historical and real-time data tends to significantly scale up the production of hydrogen by microalgae from lab scale to pilot and industrial scale. This is because the analysis of larger datasets for the identification of patterns and trends allows for more accurate predictions of hydrogen output. Otálora et al. [121] reported on the use of artificial neural networks in the characterization of the morphological differences between microalgae genera [121]. This characterization is essential to ascertain the quality of microalgae biomass, which is crucial for optimizing hydrogen production [122]. The integration of ML models into economics and economic data tends to enhance the environmental sustainability of biohydrogen production from microalgae resources. This integration tends to support techno-economic analysis (TEA) and life cycle assessment (LCA) through the provision of more comprehensive data that gives a clear economic and environmental impact of biohydrogen production from microalgae resources [123].

## Limitations and future directions

While AI and ML offer transformative potential for optimizing microalgal biohydrogen production, their practical application is currently hindered by several critical limitations. These limitations span the domains of data science, biological complexity, and engineering scale-up, creating a significant divide between promising predictions and real-world implementation [124]. A primary technical bottleneck is the issue of data quality and availability, as microalgal biohydrogen production involves complex biological processes that require precise and comprehensive data inputs. This is necessary because ML models are fundamentally data-driven, yet the field of microalgal biohydrogen suffers from a scarcity of large, high-quality datasets. Experimental studies are often conducted under disparate conditions, leading to heterogeneous data that are difficult to harmonize. This limitation makes models susceptible to overfitting, where they perform well on training data but fail to generalize to new conditions, thus reducing their predictive performance and practical utility. The complexity and nonlinear nature of biological processes further exacerbate this, as models may capture some aspects of the system but struggle to accurately predict outcomes in novel scenarios [124]. Furthermore, a significant gap exists between laboratory-scale AI model development and industrial application. While ML tools show promise for optimizing lab-scale cultivation parameters, their use in industrial settings to increase biomass and biohydrogen production is still in its infancy. This is because biological systems are inherently complex, and microalgal physiology is influenced by a multitude of interacting factors that are challenging to replicate or control at scale. Consequently, the lack of generalized protocols and standardized strategies prevents the development of universally applicable ML tools, confining their effectiveness to specific laboratory conditions rather than large-scale industrial operations [125]. A fundamental challenge limiting the integration of AI with biological mechanisms is the fact that current AI applications often function as black boxes, predicting optimal conditions without providing insights into the underlying biological pathways, such as hydrogenase activity or metabolic fluxes under stress like sulfur deprivation. To truly enhance production, future models must move beyond simple pattern recognition to incorporate biological knowledge, enabling them to not just predict but to 2022 explain and guide genetic or metabolic engineering strategies for more robust and efficient hydrogen production [126].

Another critical limitation is incomplete genomic information of many microalgae species, leading to a reduction in predictive modeling accuracy [127]. This is because AI models such as AlphaFold and Evo depend on complete genomic data to predict the structures of proteins, gene functions, and metabolic pathways [128]. The dependence on incomplete genomic information may lead to misidentification of gene functions, create gaps in pathway construction, and result in poor generalization in AI model training. This affects metabolic engineering strategies, leading to poor optimization of hydrogen-producing enzymes. Similarly, the limited genetic manipulation tools also limit the use of smart bioengineering techniques in enhancing microalgae biohydrogen potential. This is attributed to the fact that CRISPR/Cas systems and other gene editing methods are underdeveloped for many microalgae, thus precise and high-throughput genome editing.

Whereas AI tools such as OpenCRISPR and EVOLVEpro require precise genomic sequences to design highly efficient guide RNAs and optimize editing strategies, thereby maximizing editing efficiency and minimizing off-target effects. To address this issue, the use of hybrid sequencing approaches, such as combining short-read and long-read sequencing technologies, eases the generation of more complete genomic data for accurate predictive modeling. Also, the integration of experimental data with computational predictions will validate the accuracy of AI models and improve their generalization capabilities. To overcome incomplete genomic data in microalgae, integrative omics approaches combining transcriptomics, proteomics, and metabolomics can supplement missing genome information, enhancing AI predictive accuracy. Advances in long-read sequencing and pan-genome assembly will provide more complete reference genomes, improving structure and pathway predictions. Concurrently, developing versatile CRISPR/Cas systems and optimizing AI-guided tools like OpenCRISPR for microalgae will enable precise, high-throughput genome editing. Coupling these strategies allows accurate functional annotation, robust metabolic modeling, and targeted enzyme optimization, thereby enhancing biohydrogen production.

## Conclusion

Microalgae offer a sustainable platform for biohydrogen production, but biological limitations and environmental sensitivity hinder large-scale application. Smart bioengineering strategies are emerging as transformative solutions, including PSII modulation, oxygen-tolerant hydrogenase engineering, and enhanced electron flux to hydrogenases, which significantly boost hydrogen yields. Coupled with advanced bioreactors, real-time monitoring, and process optimization, these approaches create robust, high-efficiency production systems. Integrating hydrogen generation with co-product synthesis, such as biofuels, bioplastics, and high-value metabolites, enhances economic viability while aligning with circular bioeconomy principles. The fusion of synthetic biology, multi-omics analyses, and AI-driven metabolic modeling enables predictive, adaptive optimization of microalgal metabolism, accelerating the transition from lab-scale feasibility to industrial-scale implementation. By converging genetic, computational, and process innovations, smart bioengineering unlocks the full potential of microalgae, paving the way for sustainable, environmentally friendly, and economically competitive biohydrogen production platforms.

## Data Availability

No datasets were generated or analyzed during the current study.
